# Associations Between Physical Activity Measurement Modality and Gut Microbiome in Breast Cancer Survivors

**DOI:** 10.3390/nu18193286

**Published:** 2026-10-07

**Authors:** Whitney N. Neal, Keri M. Kemp, Rebecca B. Little, Stephen J. Carter, Erica A. Schleicher, Robert W. Motl, Jin Lu, Elliot J. Lefkowitz, Robert A. Oster, Lyse A. Norian, Gary R. Hunter, Helen Krontiras, Bulent Turan, Laura Q. Rogers

**Affiliations:** 1 Department of Medicine, University of Alabama at Birmingham, Birmingham, AL 35294, USA; kekemp@uabmc.edu (K.M.K.); rbarnhill@uabmc.edu (R.B.L.); jlu@uabmc.edu (J.L.); roster@uabmc.edu (R.A.O.); 2 Department of Kinesiology, Indiana University Bloomington, Bloomington, IN 47405, USA; stjcarte@iu.edu; 3 Department of Hematology Oncology, University of Pittsburgh, Pittsburgh, PA 15260, USA; eschleicher@upmc.edu; 4 Department of Kinesiology and Nutrition, University of Illinois at Chicago, Chicago, IL 60607, USA; robmotl@uic.edu; 5 Department of Computer Science, University of Alabama at Birmingham, Birmingham, AL 35294, USA; elliotl@uab.edu; 6 Markey Cancer Center, Department of Microbiology, Immunology, and Molecular Genetics, University of Kentucky, Lexington, KY 40506, USA; lyse.norian@uky.edu; 7 Department of Nutrition Sciences, University of Alabama at Birmingham, Birmingham, AL 35294, USA; ghunter@uab.edu; 8 Department of Surgery, University of Alabama at Birmingham, Birmingham, AL 35294, USA; hkrontir@uabmc.edu; 9 Department of Psychology, Koç University, Istanbul 34450, Turkey; bturan@ku.edu.tr

**Keywords:** physical activity, cardiorespiratory fitness, gut microbiome, breast cancer

## Abstract

Background/Objectives: Physical activity (PA), physiological fitness, and the gut microbiome have each been linked to health outcomes among breast cancer survivors (BCS); however, these associations have been inconsistent. Few studies have directly compared the associations of self-reported PA, accelerometer-derived PA, and physiological fitness with the gut microbiome within the same population. This study evaluated whether associations with gut microbiome outcomes vary by PA and fitness measurement modality in post-primary treatment BCS. Methods: Baseline data from 69 BCS enrolled in a randomized controlled trial were analyzed. PA was assessed using self-reported moderate-to-vigorous PA (MVPA) and accelerometer-derived light PA (LPA), MVPA, and sedentary behaviors. Physiological fitness was evaluated using peak oxygen uptake ($\dot{V}$O_2_peak) and related fitness metrics. Gut microbiome outcomes included α-diversity, β-diversity, and exploratory taxa-level analyses derived from 16S rRNA sequencing. Associations were evaluated using correlation and regression analyses, with adjustment for relevant clinical covariates. Results: Accelerometer-derived PA, particularly MVPA, demonstrated the strongest and most consistent associations with α-diversity and β-diversity, whereas physiological fitness measures showed only weak associations. Sedentary behavior was inversely associated with both diversity metrics, while self-reported MVPA was not associated with diversity indices. Medication burden and comorbidity status attenuated several PA-microbiome associations, and no taxa-level associations remained significant after correction for multiple testing. Conclusions: Associations between PA, fitness, and gut microbiome outcomes varied according to measurement modality. Accelerometer-derived MVPA demonstrated more consistent associations with microbiome diversity and composition than self-reported MVPA or physiological fitness. Future studies should incorporate multiple complementary measures of PA and fitness when examining PA–microbiome relationships.

## 1. Introduction

Breast cancer survivors (BCS) represent a rapidly growing population that often experience treatment-related sequelae including persistent fatigue [1], reduced physical function [2,3], and elevated risk of chronic comorbid conditions [4,5]. Physical activity (PA) is among the most recommended behavioral strategies for improving survivorship outcomes, with reported improvements in cardiorespiratory fitness, muscle strength, body composition, fatigue, sleep, anxiety/depression, and overall quality of life [6,7,8]. Emerging evidence also suggests that the gut microbiome may play an important role in health and quality of life in cancer survivors through its involvement in immune regulation, metabolism, and inflammation [9,10]. Gut microbiome research commonly evaluates several related but distinct outcomes, including species richness, α-diversity, β-diversity, and differential abundance of microbial taxa. Species richness refers to the number of microbial taxa identified within an individual sample, whereas α-diversity reflects both the richness and evenness of taxa within a single microbial community. In contrast, β-diversity measures differences in microbial community composition between individuals or groups and can reveal broad community-level differences associated with health characteristics, behaviors, or clinical outcomes. Differential abundance analyses complement these measures by examining whether specific microbial taxa vary according to participant characteristics or exposures, thereby identifying microbes that may contribute to observed differences in health outcomes. Together, these metrics provide insight into both overall microbial structure and the specific taxa that may influence survivorship-related outcomes, and they have been widely used to characterize associations between PA and the gut microbiome in both healthy and clinical populations [11,12].

Growing evidence from both general and clinical populations suggests that PA is associated with multiple dimensions of the gut microbiome. Several studies have reported positive associations between PA and α-diversity, β-diversity, and specific microbial taxa in healthy adults [13,14,15], indicating that physically active individuals exhibit more diverse and compositionally distinct gut microbial communities. However, findings remain inconsistent, with some studies reporting weak, selective, or null relationships with microbial diversity and community composition [16,17,18]. Similarly, higher cardiorespiratory fitness has been linked to greater microbial diversity in individuals with [19,20] and without [21] a history of breast cancer, but these findings have not been consistently replicated.

One potential explanation for these discrepancies is the variation in methods used to assess PA behavior. Questionnaires capture self-reported PA based on recalled activity over a specific time period, whereas accelerometers measure free-living movement behavior across intensity levels and sedentary time [22,23]. In contrast, physiological measures including peak oxygen uptake ($\dot{V}$O_2_peak) reflect the functional capacity to perform and adapt to PA. Unlike self-report or accelerometer-derived PA, physiological fitness is influenced by adaptations consistent with not only regular PA and sedentary behavior but also age, genetics, body composition, treatment history, and overall health status [24,25]. Therefore, measures of PA (behavior) and physiological fitness are related but distinct constructs and should not be assumed to produce equivalent microbiome associations.

Despite growing interest in PA–microbiome relationships, few studies have directly compared associations between the gut microbiome and self-reported PA, accelerometer-derived PA, and physiological fitness within the same population. As a result, it remains unclear whether observed associations with the gut microbiome reflect biological differences or inconsistent exposure measurement [11,26]. This limitation is particularly relevant among BCS, a population that often experiences persistent treatment-related effects, including metabolic dysregulation, inflammation, and reduced physical function, all of which may be influenced by both PA and the gut microbiome. Improved understanding of how different PA and fitness measures relate to the microbiome in BCS could therefore help clarify potential behavioral pathways for promoting long-term health and survivorship outcomes. PA and fitness measures are further frequently incorporated into behavioral interventions and observational studies in BCS, yet the implications of measurement selection are rarely examined.

**Figure 1:**
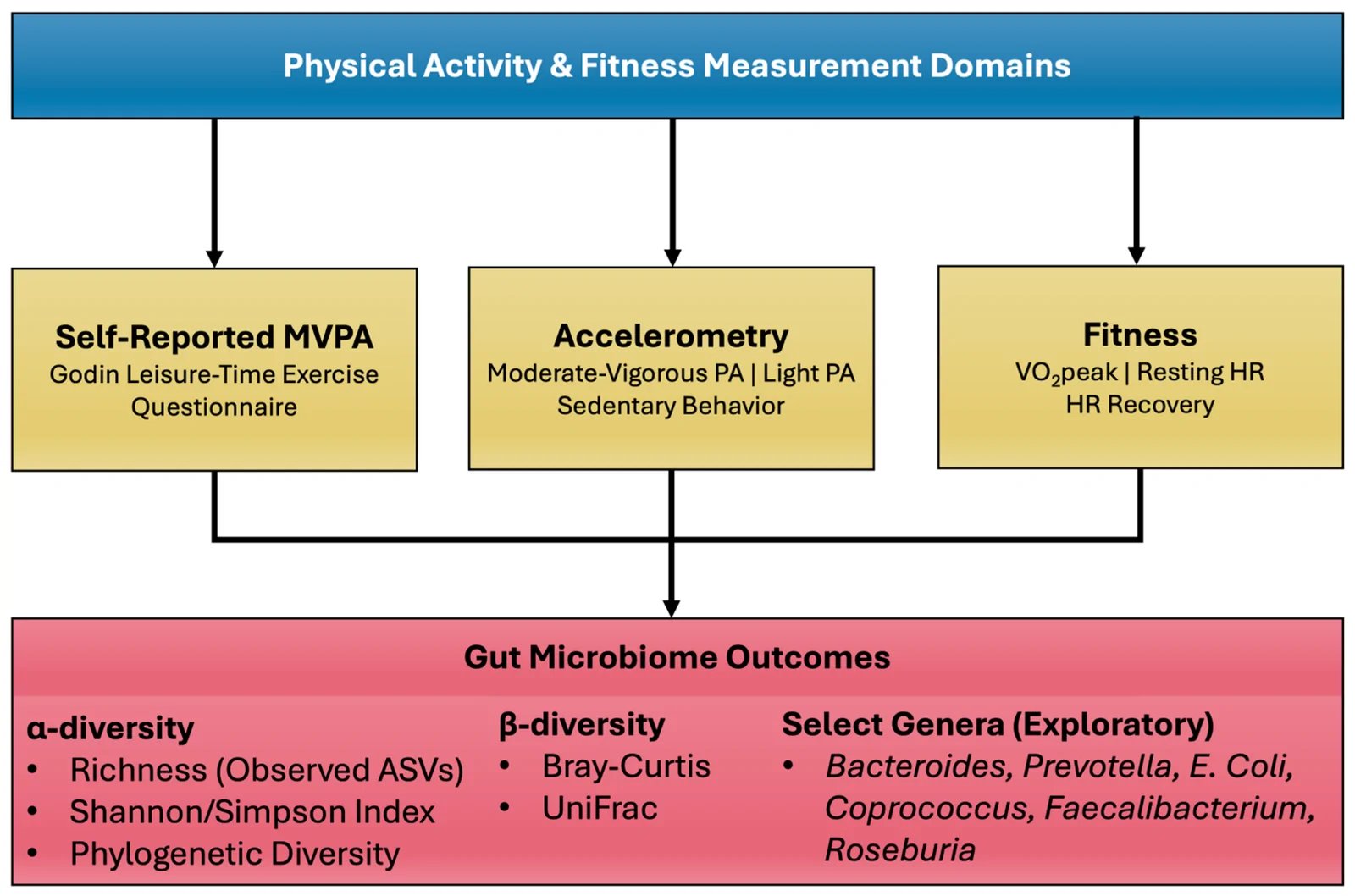
Study design and analytic framework. Baseline data from post-treatment breast cancer survivors enrolled in a randomized controlled exercise and breast cancer trial were used to examine whether associations between physical activity-related measurements and gut microbiome outcomes differ by measurement modality. MVPA = moderate-to-vigorous physical activity; PA = physical activity; HR = heart rate; ASVs = amplicon sequence variants.

The current study evaluated associations between gut microbiota diversity and self-reported PA, accelerometer-derived PA, and physiological fitness in BCS (Figure 1). We further explored associations with differential abundance (taxa-level outcomes) as a secondary objective. Based on the limited literature, we hypothesized that PA measures would demonstrate small associations with gut microbiota α-diversity, while indicators of physiologic fitness (e.g., $\dot{V}$O_2_peak) would demonstrate moderate associations [19,20,21]. Clarifying these measurement differences may improve interpretation of exercise interventions in BCS and inform selection of PA and fitness outcomes in future microbiome studies.

## 2. Materials and Methods

### 2.1. Study Design and Participants

The present study is a secondary, cross-sectional analysis of baseline data collected as part of a randomized controlled trial (RCT) in post-primary treatment BCS. The study design and recruitment strategy have been described in detail elsewhere [27]. Briefly, the Role of Gut Microbe Composition in Psychosocial Symptom Response to Exercise Training in Breast Cancer Survivors (ROME) trial examined the effects of a structured aerobic exercise intervention compared to attention control on gut microbiota composition among BCS between 18–74 years of age with self-reported fatigue (*N* = 71). Key eligibility criteria included $\dot{V}$O_2_peak <30 mL·kg^−1^·min^−1^ and completion of primary cancer treatment (≥1-year post-chemotherapy and/or radiation) as described in detail in the ROME protocol paper [27]. Participants followed an energy-balanced controlled feeding protocol prior to baseline assessments to minimize diet variability in gut microbiome outcomes. Baseline assessments were completed in the following order: completion of the controlled feeding protocol (1 week prior), $\dot{V}$O_2_peak testing, self-report surveys, and distribution of home-based measures (fecal sample collection kits and accelerometers).

All procedures for the parent RCT were conducted in accordance with the ethical guidelines set forth by the local institutional review board (University of Alabama at Birmingham/UAB IRB#30000320) and all participants provided written informed consent prior to study involvement.

### 2.2. Physical Activity and Fitness Measures

#### 2.2.1. Self-Reported Activity

A modified version of the Godin Leisure-Time Exercise Questionnaire (GLTEQ) [28,29] was used to assess weekly minutes of leisure-time PA. The modified GLTEQ, which has been widely used in breast cancer research, asks participants to report the frequency and average duration of vigorous, moderate, and light leisure-time PA performed in bouts of at least 10 min during a typical week over the previous month [30,31]. Vigorous minutes were doubled before adding to moderate minutes to derive moderate-equivalent MVPA minutes per week [32].

#### 2.2.2. Accelerometer-Measured Activity

Free-living PA was assessed using ActiGraph GT3X-BT triaxial accelerometers worn at the waist for seven consecutive days. Participants were instructed to wear the accelerometer during waking hours except while bathing, showering, or swimming, and were asked to complete an accelerometer log documenting wear time. Accelerometer data were processed in ActiLife into one-minute epochs using the low-frequency extension (LFE) filter and then scored for minute-by-minute activity counts. These counts were categorized into sedentary behavior (0–99 counts/minute), light PA (LPA) (100–1951 counts/minute), moderate PA (MPA) (1952–5724 counts/minute), and vigorous PA (VPA) (5725+ counts/minute) [33]. Wear time was validated using participant logs, and four valid days (i.e., ≥10 h of wear without ≥30 min of continuous zero counts [34]) were required to be included in the analyses. Valid data were averaged to derive weekly estimates (minutes/week) of MVPA, LPA, and sedentary behavior. As with the GLTEQ, moderate-equivalent MVPA minutes per week were calculated by weighting vigorous minutes by a factor of two before combining them with moderate minutes [32]. Sensitivity analyses using unweighted accelerometer-derived MVPA minutes per week (i.e., vigorous minutes not doubled) are also reported.

#### 2.2.3. Physiological Fitness

Resting heart rate (RHR) was measured prior to exercise testing. A graded treadmill test (Trackmaster TMX428CP; Full Vision, Newton, KS, USA) consistent with the modified Balke protocol was used to estimate $\dot{V}$O_2_peak (mL·kg^−1^·min^−1^), defined as the highest observed rate of $\dot{V}$O_2_. Workload (speed, grade) was progressively increased until volitional exhaustion, and $\dot{V}$O_2_ was assessed via indirect calorimetry and averaged over 30-s intervals. Following completion of volitional exhaustion and attainment of $\dot{V}$O_2_peak, participants immediately transitioned to an active recovery cool-down for two minutes (e.g., walking at 2.0 mph, 0% grade). Heart rate recovery (HRR) was calculated as the difference between peak heart rate and the heart rate recorded exactly 120 s (2 min) post-exercise, with a more pronounced recovery indicative of parasympathetic reactivation and ongoing sympathetic withdrawal.

### 2.3. Gut Microbiota Diversity and Composition

#### 2.3.1. Sample Collection and Sequencing

To reduce dietary variability in microbiome profiles, all stool samples were collected following completion of a standardized controlled feeding protocol for at least one week prior to sample collection. Stool samples were collected and processed as previously described [27,35]. Briefly, stool samples were collected at home in provided Para-Pak vials (Meridian Biosciences; Cincinnati, OH, USA) and shipped overnight to the Microbiome Core Laboratory at the University of Alabama at Birmingham. All samples were aliquoted into labelled cryovials and stored at −80 °C until time for DNA extraction and 16S rRNA gene sequencing using the Illumina MiSeq platform. Sequencing generated 250-bp paired-end reads, which were quality filtered before paired-end read merging and downstream analysis.

#### 2.3.2. Bioinformatics and Data Processing

Quality control, generation of amplicon sequence variants (ASVs), and taxonomic assignment were conducted as described previously [35]. In brief, sequences classified as unknown, mitochondria, or chloroplasts were removed prior to downstream analyses. Rare ASVs were subsequently filtered using the genefilter package (version 1.94.0) by retaining only those with more than two reads in at least two samples, thereby reducing the influence of potential PCR and sequencing artifacts. This filtering step reduced the number of ASVs from 5902 to 1138 while removing less than 1% of the total sequence reads. To mitigate biases associated with unequal library sizes, the full longitudinal dataset was further rarefied prior to subsetting to baseline samples. Samples were rarefied to an even depth corresponding with the minimum sequencing effort observed across the entire longitudinal dataset (19,142 reads; original range: 19,142 to 128,983 reads) using the phyloseq (version 1.56.0) function rarefy_even_depth [36] in RStudio (version 2026.05.0+218). Rarefaction was chosen over alternative normalization methods because it provides robust performance for α-diversity analyses in datasets of this size and avoids challenges associated with other approaches such as the centered log-ratio (CLR) transformation, which requires additional handling of zero-count taxa prior to analysis [37]. Because the rarefaction threshold was matched to the lowest-depth sample, all collected samples (*N* = 69) were retained for downstream analyses. Four measures of within-sample microbial α-diversity: observed species (number of taxa present), Shannon Index and Simpson Index (which account for taxon abundance and evenness), and whole-tree phylogenetic diversity (which incorporates evolutionary relatedness among taxa as a marker of trait/functional diversity), were estimated using this rarefied feature table in the phyloseq package [36].

Compositional differences (i.e., β-diversity) were calculated based on the Bray-Curtis dissimilarity [38] and weighted/unweighted UniFrac [39] using the rarefied feature table. For β-diversity, PA and fitness variables were dichotomized as above versus below the median to facilitate equal sample sizes for the comparisons. Because a substantial proportion of participants reported no leisure-time MVPA (*n* = 39), self-reported MVPA was dichotomized as 0 versus >0 min/week. Principal coordinates analyses (PCoA) of the Bray-Curtis dissimilarity and weighted/unweighted UniFrac were performed with the ape package (version 5.8-1) [40] and plotted with ggplot2 (version 4.0.3) to visualize β-diversity differences among PA and physiological fitness measures. Statistical significance of the β-diversity relationships was then evaluated with permutational multivariate analysis of variance (PERMANOVA) using the vegan function adonis2 (version 2.7-5) [41] with 9999 permutations per run.

### 2.4. Potential Covariates

Key demographic and clinical characteristics with potential to confound associations between PA, fitness, and the gut microbiome were assessed via self-administered surveys and included age, race/ethnicity, cancer-related factors (time since diagnosis, cancer stage, and treatment), number of current medications (including over the counter medications), number of comorbidities [42], any antibiotics over the last 6 months, any steroid medications or injections over the last 6 months, and menopausal status [43]. Percent body fat measured by dual-energy x-ray absorptiometry (DXA) [27] was also included due to its relevance to both PA and gut microbiota composition [44].

### 2.5. Statistical Analyses

For the present study, cross-sectional analyses used pooled baseline data from both study arms. Although 71 participants provided baseline data for at least one outcome, one participant did not provide a stool sample and accelerometer data files from two participants were unavailable. To maximize use of available data, all observations were retained for the pair-wise analysis; therefore, the analytic sample varied between 66 and 69 participants depending on the presence or absence of PA and fitness variables. Continuous variables are summarized as mean ± standard deviation (SD), and categorical variables are summarized using frequencies (*n*) and percentages (%). All analyses were performed using SAS (version 9.4) and RStudio (version 2026.5.1.225) [45]. The threshold for statistical significance was set a priori as a two-sided *p*-value < 0.05; for analyses involving multiple comparisons, false discovery rate (FDR) correction was applied using the Benjamini-Hochberg procedure [46], with statistical significance defined as *q* ≤ 0.05. Normality of PA, fitness, and gut microbiome α-diversity metrics were examined using histograms and the Shapiro–Wilk test. Because several measures exhibited skewed distributions (i.e., Simpson Index, self-reported MVPA, accelerometer-measured MVPA, sedentary behavior), nonparametric methods were used where appropriate.

For the primary analyses, Spearman’s rank-order correlations (*ρ*) were performed to estimate associations between PA, physiological fitness, and gut microbiome α-diversity outcomes, including observed species richness, Shannon diversity, Simpson diversity, and phylogenic diversity (PD). Spearman correlations were also used to evaluate associations between demographic and clinical characteristics identified *a priori* as potential confounders (see above, Section 2.4 Potential Covariates), PA and fitness measures, and α-diversity outcomes. Variables associated with both the exposure of interest (PA or fitness measure) and the microbiome outcome were considered potential confounders and included as covariates in adjusted models.

Statistically significant associations identified in correlation analyses were further examined using multivariable linear regression models, with α-diversity metrics specified as dependent variables and PA/fitness measurement modality as independent variables. Separate models were fit for each PA/fitness measurement modality and α-diversity association. These adjusted models incorporated relevant confounders identified during covariate screening as independent variables. Model assumptions were evaluated through inspection of regression diagnostics, including assessment of residual distributions.

Exploratory taxa-level associations were also examined as secondary analyses to characterize modality-specific associations between PA, fitness, and microbial taxa abundance. Taxa were identified a priori from previous studies in breast cancer survivors reporting statistically significant associations between fitness-related outcomes and the genera *Bacteroides*, *Prevotella*, *Escherichia-Shigella*, *Coprococcus*, *Faecalibacterium*, and *Roseburia* [19,20].

## 3. Results

### 3.1. Overview

Table 1 provides an overview of demographic and clinical characteristics for participants who provided fecal samples at baseline and were included in the final analytic sample (*N* = 69). Just over half of participants were White/Caucasian (54.4%), with a mean of approximately 6 years (73.6 months) since cancer diagnosis and a majority having undergone chemotherapy (47.8%) and/or radiation (69.6%).

Descriptive statistics for study variables of interest are provided in Table 2. Correlations across measurement modalities were modest, indicating limited convergence between self-reported MVPA, accelerometer-derived activity, and physiological fitness (Table A1). Self-reported MVPA showed only a small association with accelerometer-derived MVPA, whereas accelerometer MVPA demonstrated stronger relationships with key fitness indicators. Regarding covariates/potential confounders, the number of medications was inversely associated with accelerometer-derived MVPA (*ρ* = −0.24, *p* < 0.05) and both Shannon (*ρ* = −0.33, *p* < 0.01) and Simpson (*ρ* = −0.36, *p* < 0.01) diversity. Number of comorbidities was negatively associated with Shannon diversity (*ρ* = −0.25, *p* = 0.04) and accelerometer-measured LPA (*ρ* = −0.34, *p* < 0.01). No other demographic or clinical variables were significantly related to α-diversity indices.

### 3.2. α-Diversity

Associations between fitness measures and *α*-diversity were generally weak, whereas PA measures showed more consistent associations with *α*-diversity (Table 3). Accelerometer-measured sedentary behavior was negatively associated with Shannon (*ρ* = −0.28, *p* = 0.02) and Simpson (*ρ* = −0.29, *p* = 0.02) diversity. There was a significant moderate positive correlation between accelerometer-derived LPA and observed species (*ρ* = 0.31, *p* = 0.01). Accelerometer-measured MVPA moderate-equivalent minutes exhibited positive associations with Shannon (*ρ* = 0.39, *p* < 0.001) and Simpson (*ρ* = 0.39, *p* = 0.001) diversity, while self-reported MVPA was not associated with any diversity indices. After correction for multiple comparisons, only the relationship between accelerometer-derived MVPA with Shannon and Simpson remained statistically significant (*q* = 0.007 for Shannon; *q* = 0.006 for Simpson).

As a sensitivity analysis, a GLTEQ Health Contribution Score (HCS) was calculated by weighting weekly frequencies of moderate and vigorous PA according to their metabolic equivalent (MET) values and summing the resulting scores [28]. These analyses did not change the results. Additional sensitivity analyses using unweighted accelerometer-measured MVPA (i.e., vigorous minutes not doubled) with Shannon and Simpson diversity remained virtually unchanged (*ρ* = 0.39 for both).

In unadjusted regression analyses, accelerometer-derived MVPA moderate-equivalent minutes was positively associated with Shannon diversity F(1, 65) = 6.07, *p* = 0.01, and Simpson diversity F(1, 65) = 6.50, *p* = 0.01, accounting for ≈7–8% of the variance in diversity indices. These associations were attenuated after adjustment for covariates, including number of medications and comorbidity burden, suggesting potential confounding (Table 4 and Table 5). Similarly, LPA was associated with Shannon diversity in unadjusted models F(1, 65) = 5.20, *p* = 0.03, but this relationship was no longer statistically significant after accounting for number of medications and comorbidity burden (Table 6). Sensitivity analyses using unweighted accelerometer-measured MVPA (i.e., vigorous minutes not doubled) and generalized linear models yielded similar results.

### 3.3. β-Diversity

**Scheme 1:**
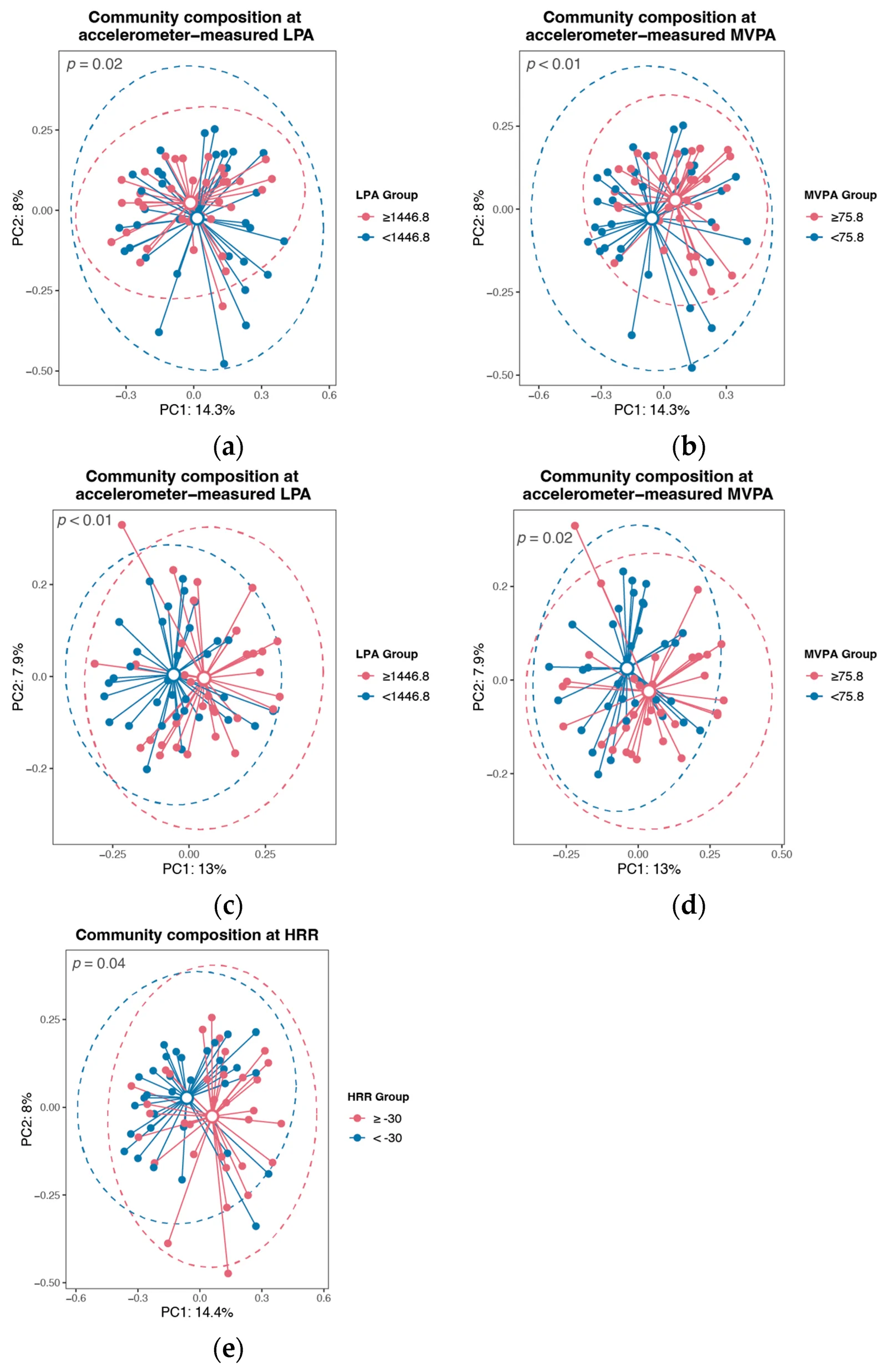
Principal coordinates analyses (PCoA) demonstrating differences in community composition by physical activity modality. (**a**) Accelerometer-measured light physical activity, defined by median split (LPA; <1446.8 vs. ≥1446.8 min/week) (Bray-Curtis). (**b**) Accelerometer-measured moderate-to-vigorous physical activity, defined by median split (MVPA; <75.83 vs. ≥75.83 min/week) (Bray-Curtis). (**c**) Accelerometer-measured LPA, defined by median split (unweighted UniFrac). (**d**) Accelerometer-measured MVPA, defined by median split (unweighted UniFrac). (**e**) Heart rate recovery, defined by median split (HRR; <−30 bpm vs. ≥−30 bpm (Bray-Curtis). Points represent individual participants and dashed ellipses represent 95% confidence intervals surrounding group centroids.

For β-diversity analyses, continuous fitness and PA variables were dichotomized as described in the Methods. Among fitness measures, significant differences in community composition were identified for HRR based on Bray-Curtis dissimilarity (*p* = 0.04; Scheme 1e). No statistically significant differences in overall microbial composition were observed for any other fitness measures.

In contrast, accelerometer-derived PA measures were associated with significant differences in microbial community composition (Scheme 1a–d). Moderate-equivalent minutes per week of accelerometer-measured MVPA were associated with multiple β-diversity distance metrics, including Bray-Curtis dissimilarity (*p* < 0.01) and unweighted UniFrac (*p* = 0.02). Similarly, LPA minutes per week were associated with β-diversity using Bray–Curtis (*p* = 0.02) and unweighted UniFrac (*p* < 0.01). These outcomes remained statistically significant after adjustment for covariates and correction for multiple comparisons. Sedentary behavior and self-reported moderate-equivalent MVPA minutes/week were not associated with β-diversity across any metric. Sensitivity analyses using unweighted accelerometer-measured MVPA and the GLTEQ HCS yielded similar results.

**Scheme 2:**
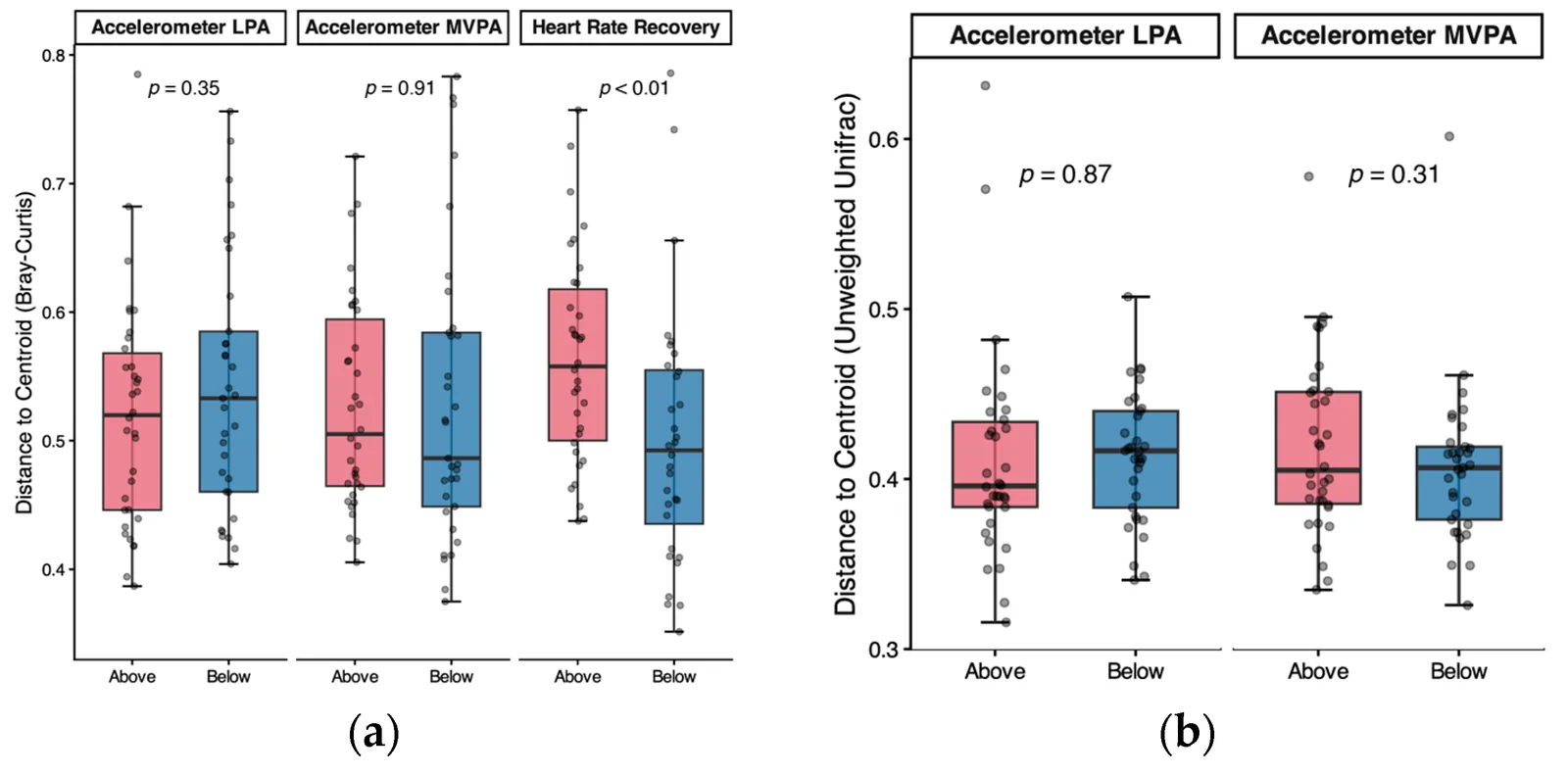
Multivariate dispersion of gut microbial community composition by physical activity modality. (**a**) Distance to group centroid based on Bray–Curtis dissimilarity for participants categorized above or below the median for accelerometer-measured light physical activity (LPA; <1446.8 vs. ≥1446.8 min/week; *p* = 0.35), moderate-to-vigorous physical activity (MVPA; <75.8 vs. ≥75.8 min/week; *p* = 0.91), and heart rate recovery (HRR; <−30 bpm vs. ≥−30 bpm; *p* < 0.01). (**b**) Distance to group centroid based on unweighted UniFrac distances for participants categorized above or below the median for accelerometer-measured LPA (*p* = 0.87) and MVPA (*p* = 0.31). Boxplots display the median, interquartile range, and 1.5× interquartile range; points represent individual participants. Greater distance-to-centroid values indicate greater within-group variability in microbial community composition.

To evaluate whether these differences reflected true shifts in community composition or unequal within-group variance, multivariate dispersion was assessed using PERMDISP. Dispersion did not differ between accelerometer-derived MVPA (Bray-Curtis: *p* = 0.91; unweighted UniFrac: *p* = 0.31) or LPA (Bray-Curtis: *p* = 0.35; unweighted UniFrac: *p* = 0.87) groups, supporting the interpretation that observed β-diversity differences were driven by shifts in microbial community composition (Scheme 2). Conversely, HRR groups exhibited significantly different dispersion (*p* < 0.01), suggesting that the associated Bray-Curtis results may be influenced by greater within-group heterogeneity (Scheme 2a).

### 3.4. Differential Taxon Abundance

**Scheme A1:**
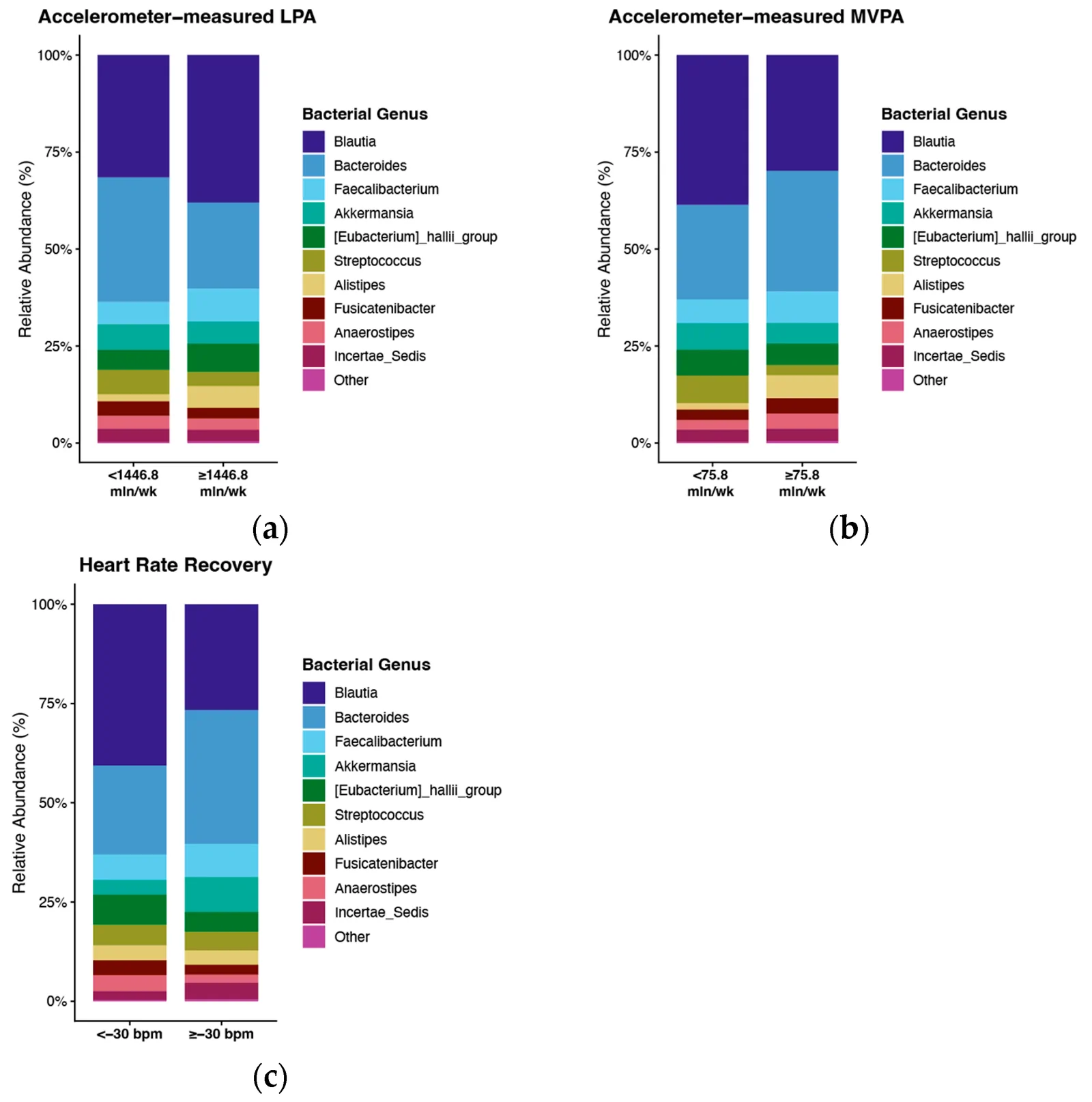
Top 10 genera truncated for each measure of physical activity. (**a**) Accelerometer-derived light physical activity (LPA), defined by median split (<1446.8 vs. ≥1446.8 min/week). (**b**) Accelerometer-derived moderate-to-vigorous physical activity (MVPA), defined by median split (<75.83 vs. ≥75.83 min/week). (**c**) Heart rate recovery, defined by median split (<−30 vs. ≥−30 bpm). Stacked bar plots depict the relative abundance of the 10 most abundant genera within each group. Only physical activity modalities demonstrating statistically significant β-diversity associations after adjustment for covariates and correction for multiple comparisons are shown.

The relative abundance of the 10 most abundant genera by group is shown in Scheme A1. Exploratory taxa-level analyses revealed no statistically significant associations between fitness measures and the abundance of selected genera (*Bacteroides*, *Prevotella*, *Escherichia*-*Shigella*, *Coprococcus*, *Faecalibacterium*, *Roseburia*). Among PA measures, accelerometer-derived LPA was associated with *Roseburia* abundance (*ρ* = 0.26, *p* = 0.04); however, these associations were no longer statistically significant after adjustments were made for multiple comparisons.

## 4. Discussion

The present study examined whether associations between PA, physiological fitness, and gut microbiome diversity vary by measurement modality among post-primary treatment breast cancer survivors. Contrary to our initial hypothesis, fitness measures exhibited generally weak and inconsistent associations with gut microbiota diversity, with only modest associations observed for HRR and β-diversity. By comparison, accelerometer-derived PA showed the most consistent relationships with both α-diversity and β-diversity metrics, whereas self-reported MVPA was not associated with any diversity indices. Collectively, these findings suggest that the strength and consistency of PA-microbiome associations vary by measurement modality among post-primary treatment, non-metastatic BCS.

Recent large-scale studies in healthy populations reported associations between accelerometer-derived PA behaviors and microbial diversity and functional pathways, although the specific microbiome outcomes vary across populations and study designs [11,15,47,48,49,50]. Consistent with these findings, accelerometer-derived moderate-equivalent MVPA, LPA, and sedentary behavior demonstrated significant associations with α-diversity indices incorporating community richness and evenness, while MVPA and LPA were the modalities most consistently related to β-diversity [15,48,50]. Notably, accelerometer-derived MVPA was associated with multiple β-diversity distance measures, including Bray-Curtis and weighted UniFrac, suggesting that objectively measured MVPA may be associated with both within-sample microbial diversity and broader differences in community composition.

Conversely, self-reported MVPA was not associated with species richness (observed species) or indices incorporating both richness and evenness. These findings are concordant with the broader observation that self-reported and device-based activity measures capture related but non-equivalent dimensions of movement behavior [51], and further support the premise that measurement modality influences the magnitude and interpretation of microbiome associations.

Contrary to expectations, physiological fitness measures were largely unrelated to microbiome diversity or composition, with differences in community composition based only on HRR groups. These findings differ from prior work suggesting moderate associations between $\dot{V}$O_2_peak and gut microbiome diversity among breast cancer survivors [19,20] and other adult populations [21]. One potential explanation is the study requirement for low fitness (i.e., higher levels of fitness were not represented in our sample), yet variability in $\dot{V}$O_2_peak in our sample was similar to those reported in previous studies (SD: 4.0–5.6 mL·kg^−1^·min^−1^) [19,20,21], suggesting that relationships between cardiorespiratory fitness and the gut microbiome may be more modest or context-dependent than previously reported. Also, the controlled feeding protocol completed during the week preceding fecal sample collection may have better isolated the fitness-microbiome association independent of dietary intake, thus attenuating relationships attributable to diet and exercise performance interactions [52]. Further, differences in microbiome processing and analytical approaches across studies, including sequence filtering, diversity estimation, and statistical modeling procedures, may have also contributed to inconsistencies in observed fitness-microbiome associations. Future studies that include broader ranges of fitness and functional capacity are needed to clarify the extent to which physiological adaptation independently relates to microbial diversity and composition in this population.

With respect to taxa-level outcomes, the observed exploratory relationship between *Roseburia* abundance and LPA was attenuated after correction for multiple testing. The overall findings suggest that associations between PA, fitness, and specific microbial taxa were weaker and less consistent than those observed for global diversity metrics; however, these results should be interpreted with caution and viewed as hypothesis-generating. Future studies with larger samples and greater statistical power are necessary to determine whether specific taxa consistently respond to behavioral or physiological dimensions of PA.

Clinical characteristics emerged as important considerations when interpreting PA-microbiome relationships. Although medication burden and comorbidity burden demonstrated significant associations with both activity measures and diversity indices, medication burden appeared to be particularly relevant in the present study. Number of medications was associated with both microbial diversity and accelerometer-derived MVPA, and adjustment for medication burden attenuated several initially significant PA-microbiome associations. This finding is consistent with evidence that medication use is an important determinant of gut microbial composition [53] and suggests that medication burden may partially account for observed relationships between lifestyle behaviors and the microbiome. Because BCS are at increased risk of chronic comorbidity leading to increased medication use, medication burden may reflect both the direct influence of pharmacologic exposures on the gut microbiome and broader differences in underlying health status [54]. Thus, burden of comorbidities and number of medications warrant careful consideration in future research and may help explain variability in PA-microbiome relationships across BCS studies.

To our knowledge, this is among the first studies to simultaneously evaluate self-reported activity, accelerometer-derived activity, and physiological fitness within the same cohort, allowing direct comparison of modality-specific associations with the gut microbiome. Additional strengths include the use of objective accelerometer-based measures, the standardized controlled feeding protocol, and evaluation of multiple α-diversity and β-diversity outcomes in an understudied population of breast cancer survivors. Nonetheless, several limitations should be considered when interpreting these findings. First, the cross-sectional design precludes causal inference, and residual confounding remains possible despite adjustment for selected clinical covariates. Although accelerometers provide more objective assessments of free-living movement behavior, activity intensity was determined using standardized cut points [33] that were neither age-adjusted nor calibrated to each participant’s measured oxygen consumption. As a result, the accelerometer-derived MVPA and LPA estimates may not accurately reflect the physiological intensity of activity for all participants, potentially influencing observed modality-specific associations [55,56]. In addition, accelerometer monitoring captured activity during a relatively short assessment period and may not reflect long-term PA patterns that could be more relevant to gut microbial diversity and composition. Participants were required to have low cardiorespiratory fitness in the parent RCT; however, eligibility was not based on physical inactivity and participants demonstrated substantial variability in baseline physical activity levels. Although larger than previous microbiome studies in breast cancer survivors, the sample size may still have limited statistical power to detect modest associations. Furthermore, while the α-diversity, β-diversity, and taxa-level analyses provide valuable insight into microbial community structure, they provide only a partial view of the gut microbiome. Microbial functional outcomes (e.g., short-chain fatty acid concentrations) were not examined and may represent important pathways linking PA, fitness, and gut microbial metabolic function; therefore, the biological significance of these associations should be interpreted cautiously. Future studies employing larger, more diverse cohorts, longitudinal designs, and multi-omics approaches are warranted.

The present findings generate several hypotheses for future study. First, associations between PA and the gut microbiome may differ depending on the measurement modality used, with device-based measures potentially providing greater sensitivity for detecting microbiome-related movement behaviors than self-report or fitness measures. Second, behavioral activity and physiological fitness demonstrated different patterns of association with gut microbiome diversity, warranting additional functional and taxonomic analyses to determine whether they reflect distinct biological pathways. Third, clinical characteristics such as medication burden and comorbidity status may modify observed PA–microbiome relationships and should be routinely evaluated in future studies. Collectively, these findings suggest that PA-microbiome relationships are likely multidimensional and shaped by methodological, biological, and clinical factors that warrant further investigation.

## 5. Conclusions

This study examined whether associations between PA behavior and physiological fitness with gut microbial diversity were influenced by measurement modality in post-primary treatment breast cancer survivors. Contrary to our initial hypothesis, physiological fitness measures demonstrated few associations with gut microbiome diversity and composition. Instead, accelerometer-derived PA, particularly MVPA, exhibited the strongest and most consistent relationships with both α-diversity and β-diversity outcomes. From a clinical and translational perspective, free-living MVPA may represent a more relevant behavioral target for understanding exercise-related variation in the gut microbiome than physiological fitness alone. These findings further suggest that observed PA–microbiome relationships may be highly dependent on how PA is measured, and that self-report, accelerometer-derived activity, and physiological fitness should not be considered interchangeable exposures in microbiome research. Future studies should incorporate multiple complementary measures of PA and fitness to clarify the behavioral and physiological pathways linking movement behaviors with gut microbial health.

## Figures and Tables

**Table 1:** Participant demographic and clinical characteristics.

	n	*N* (%)
Age (yr) (mean ± SD)	69	57 ± 10
Race (n, %)	68	
White/Caucasian		37 (54.4)
Black/African American		31 (45.6)
Cancer Status		
Months since diagnosis (mean ± SD)	69	73.6 ± 63.7
Cancer stage (n, %)	69	
DCIS		9 (13)
I		28 (40.6)
II		24 (34.8)
III		8 (11.6)
History of chemotherapy (yes)	69	33 (47.8)
History of radiation (yes)	69	48 (69.6)
Current hormone treatment (yes)	68	35 (51.5)
Menopausal status (yes)	66	56 (84.8)
Body mass index, kg/m^2^ (mean ± SD)	69	32.5 ± 7.5
Body fat, %	69	46.7 ± 5.2
Current number of medications	69	4.4 ± 3.0

**Table 2:** Descriptive statistics for physical activity, physiological fitness, and α-diversity indices.

	*M* ± *SD*	Range
Physical activity measures, min/week		
Accelerometer Sedentary	4930.0 ± 1838.0	1624.0 to 10,339.0
Accelerometer LPA	1462.0 ± 553.8	355.5 to 3194.5
Accelerometer MVPA	112.7 ± 109.9	7.0 to 614.0
Self-report MVPA ^1^	75.1 ± 157.7	0 to 900.0
Fitness measures		
Resting heart rate (RHR), bpm	76 ± 12	52 to 106
$\dot{V}$O_2_peak, mL·kg^−1^·min^−1^	19.3 ± 4.1	10.4 to 27.2
Heart rate recovery (HRR), bpm	−30 ± 10	−55 to −6
α-diversity, a.u.		
Observed species	168.6 ± 48.2	48.0 to 296.0
Shannon	3.4 ± 0.6	2.1 to 4.7
Simpson	0.9 ± 0.1	0.6 to 1.0
PD (whole tree)	9.0 ± 2.2	4.4 to 15.8

Note: LPA = light physical activity, MVPA = moderate-to-vigorous physical activity, PD = phylogenetic diversity. ^1^ Based on the modified Godin Leisure-Time Exercise Questionnaire.

**Table 3:** Spearman’s rank-order correlation coefficients for physical activity, physiological fitness, and α-diversity.

	N	Shannon	Simpson	Observed	Phylogenetic
Accelerometer sedentary	67	−0.28 *	−0.29 *	−0.04	0.00
Accelerometer LPA	67	0.24	0.19	0.31 *	0.24
Accelerometer MVPA	67	0.39 **	0.39 **	0.20	0.11
Self-Report MVPA ^1,2^	69	0.08	0.02	0.20	0.18
Resting heart rate (RHR)	69	0.05	0.09	0.05	0.04
$\dot{V}$O_2_peak	69	0.20	0.13	0.18	0.12
Heart rate recovery (HRR)	66	−0.07	−0.08	0.01	0.07

Note: LPA = light physical activity, MVPA = moderate-to-vigorous physical activity. ^1^ Based on the modified Godin Leisure-Time Exercise Questionnaire. ^2^ Data capped at mean + 2 SD prior to statistical analysis to improve estimate precision and reduce the potential bias of extreme values. * *p* < 0.05; ** *p* < 0.01.

**Table 4:** Modeling Shannon Index adjusted for accelerometer-derived MVPA, medications, and comorbidities (*n* = 66).

	Adjusted *R*^2^	Std. *β*	Partial *r*	95% CI
Model 1: Shannon	0.07			
Accelerometer MVPA		0.29 *		0.06, 0.53
Model 2: Shannon	0.17			
Accelerometer MVPA		0.20	0.10	−0.03, 0.43
# medications		−0.35 **	0.14	−0.61, −0.09
# comorbidities		−0.03	<0.001	−0.30, 0.23

* *p* < 0.05; ** *p* < 0.01.

**Table 5:** Modeling Simpson Index adjusted for accelerometer-derived MVPA, medications, and comorbidities (*n* = 66).

	Adjusted *R*^2^	Std. *β*	Partial *r*	95% CI
Model 1: Simpson	0.08			
Accelerometer MVPA		0.30 *		0.07, 0.54
Model 2: Simpson	0.27			
Accelerometer MVPA		0.19	0.11	−0.03, 0.41
# medications		−0.48 **	0.23	−0.73, −0.23
# comorbidities		0.02	<0.001	−0.22, 0.27

* *p* < 0.05; ** *p* < 0.01.

**Table 6:** Modeling Shannon Index adjusted for accelerometer-derived LPA, medications, and comorbidities (*n* = 66).

	Adjusted *R*^2^	Std. *β*	Partial *r*	95% CI
Model 1: Shannon	0.06			
Accelerometer LPA		0.27 *		0.03, 0.51
Model 2: Shannon	0.16			
Accelerometer LPA		0.18	0.08	−0.06, 0.42
# comorbidities		−0.01	<0.001	−0.28, 0.26
# medications		−0.37 **	0.14	−0.63, −0.10

* *p* < 0.05; ** *p* < 0.01.

## Data Availability

The raw sequence data generated in this study have been deposited in the National Center for Biotechnology Information (NCBI) Sequence Read Archive (SRA) under BioProject accession number PRJNA1536543 and BioSample accession numbers SAMN63645315 to SAMN63645554. All other data supporting the conclusions of this article will be made available by the authors upon reasonable request and after full execution of a data use agreement according to our institution’s policies and Institutional Review Board requirements.

## Abbreviations

The following abbreviations are used in this manuscript:

BCS	Breast cancer survivors
PA	Physical activity
RCT	Randomized controlled trial
ROME	Role of Gut Microbe Composition in Psychosocial Symptom Response to Exercise Training in Breast Cancer Survivors
UAB	University of Alabama at Birmingham
GLTEQ	Godin Leisure-Time Exercise Questionnaire
MVPA	Moderate-to-vigorous physical activity
LPA	Light physical activity
RHR	Resting heart rate
HRR	Heart rate recovery
ASV	Amplicon sequence variants
PD	Phylogenetic diversity
PCoA	Principle coordinates analyses
PERMANOVA	Permutational multivariate analysis of variance
DXA	Dual-energy x-ray absorptiometry
FDR	False discovery rate

## Appendix A

**Table A1:** Correlation matrix among physical activity and physiological fitness measurement modalities. Matrix displaying pairwise Spearman correlation coefficients among self-reported moderate-to-vigorous physical activity, accelerometer-derived physical activity and sedentary behavior, and physiological fitness measures in post-treatment breast cancer survivors. LPA = light physical activity, MVPA = moderate-to-vigorous physical activity, RHR = resting heart rate, HRR = heart rate recovery.

	1	2	3	4	5	6	7
1. Accelerometer sedentary	–						
2. Accelerometer LPA	−0.23 *	–					
3. Accelerometer MVPA	−0.39 **	0.30 *	–				
4. Self-Report MVPA ^1,2^	−0.02	0.08	0.25 *	–			
5. RHR	0.06	−0.02	−0.11	−0.01	–		
6. $\dot{V}$O_2_peak	−0.15	0.26 *	0.40 **	0.26 *	−0.16	–	
7. HRR	−0.01	0.07	−0.11	0.02	0.17	−0.07	–

Note: LPA = light physical activity, MVPA = moderate-to-vigorous physical activity. ^1^ Based on the modified Godin Leisure-Time Exercise Questionnaire. ^2^ Data capped at mean + 2 SD prior to statistical analysis to improve estimate precision and reduce the potential bias of extreme values. * *p* < 0.05; ** *p* < 0.01.
